# Autism spectrum symptoms in children with neurological disorders

**DOI:** 10.1186/1753-2000-6-34

**Published:** 2012-11-12

**Authors:** Hilde K Ryland, Mari Hysing, Maj-Britt Posserud, Christopher Gillberg, Astri J Lundervold

**Affiliations:** 1 Department of Biological and Medical Psychology, University of Bergen, Bergen, Norway; 2 Centre for Child and Adolescent Mental Health and Welfare, Uni Research, Bergen, Norway; 3 Department of Child and Adolescent Psychiatry, Haukeland University Hospital, Bergen, Norway; 4 Gillberg Neuropsychiatry Centre, Sahlgrenska Academy, University of Gothenburg, Gothenburg, Sweden

**Keywords:** Children, Neurological disorders, Autism spectrum symptoms, The Autism Spectrum Screening Questionnaire

## Abstract

**Background:**

The aims of the present study were to assess symptoms associated with an autism spectrum disorder (ASD) in children with neurological disorders as reported by parents and teachers on the Autism Spectrum Screening Questionnaire (ASSQ), as well as the level of agreement between informants for each child.

**Methods:**

The ASSQ was completed by parents and teachers of the 5781 children (11–13 years) who participated in the second wave of the Bergen Child Study (BCS), an on-going longitudinal population-based study. Out of these children, 496 were reported to have a chronic illness, including 99 whom had a neurological disorder. The neurological disorder group included children both with and without intellectual disabilities.

**Results:**

Children with neurological disorders obtained significantly higher parent and teacher reported ASSQ scores than did non-chronically ill children and those with other chronic illnesses (*p*<.01; ES = .50-1.01), and 14.1% were screened above the positive cutoff score for ASD according to their combined parent and teacher ASSQ scores. Parent/teacher agreement over ASSQ scores for children with neurological disorders was moderate to high for the total score and for three sub scores generated from a factor analysis, and low to moderate for single items.

**Conclusions:**

The ASSQ identifies a high rate of ASD symptoms in children with neurological disorders, and a large number of children screened in the positive range for ASD. Although a firm conclusion awaits further clinical studies, the present results suggest that health care professionals should be aware of potential ASD related problems in children with neurological disorders, and should consider inclusion of the ASSQ or similar screening instruments as part of their routine assessment of this group of children.

## Background

Children with neurological disorders are reported to have a high rate of problems associated with autism spectrum disorders (ASD), as well as an increased risk of receiving an ASD diagnosis [1]. ASD refers to a group of developmental/psychiatric disorders characterized by severe social and communication difficulties, as well as by a restricted pattern of repetitive and stereotyped behaviors [2]. For the present study, we will use the Autism Spectrum Screening Questionnaire (ASSQ) [3] to assess symptoms associated with ASD in a population based sample of children with neurological disorders.

Previous studies assessing ASD symptoms in children with specific neurological disorders have used a range of different assessment methods. One recent study showed that at 24 months of age, prematurely born children diagnosed with cerebral palsy were more likely than preterm babies without this diagnosis to screen positively for ASD on the Modified Checklist for Autism in Toddlers (M-CHAT) [4], when assessed at 24 months of age [5]. In studies of hydrocephalus, ASD as measured by the Autistic Behavior Checklist (ABC) [6] were present in 23% of children aged 6–17 [7], and autism as rated by the Childhood Autism Rating Scale (CARS) [8] was reported in 13% of children aged 5–12 [9]. In a study using the parent version of the Autism Screening Questionnaire (ASQ) in assessing symptoms of ASD [10], 32% of children aged 2–18 with epilepsy included from a tertiary care epilepsy clinic were found to fulfill the ASQ criteria for having an ASD [11]. In a questionnaire-based study using parent report, 3.1% of males with Duchenne Muscular Dystrophy were reported to have an ASD [12].

Other clinical studies have used diagnostic instruments to estimate prevalence rates of clinical diagnoses within the autism spectrum. According to the Autism Diagnostic Interview-Revised (ADI-R) [13], 49% of children under the age of 18 with myotonic dystrophy type 1 had an ASD [14], while 15% of children aged 4–18 with cerebral palsy have been reported to meet DSM-IV criteria for an ASD [15].

In spite of an increasing awareness in child psychiatry and developmental medicine that various conditions, such as neurological disorders and ASD, often co-exist and share symptoms [16-19], health services for children largely remain specialized. Children with neurological disorders are most commonly treated by specialist somatic health care services in pediatric clinics, where psychiatric problems, such as ASD, may remain unrecognized [19,20]. A study from 2006 showed that only 8% of pediatricians routinely screened for ASD, partly due to unfamiliarity with ASD screening tools [21]. Awareness and use of adequate screening instruments to identify symptoms of ASD would thus be of benefit to pediatric clinics.

The ASSQ [3] has, to our knowledge, not previously been used in studies focusing on children with neurological disorders. The questionnaire has been validated as a screening tool for ASD symptoms by studies in northern Europe and UK since the late 1990s [3,22-26], more recently in China [27], and in Norway using data from the first wave of the Bergen Child Study (BCS) [28]. The BCS sub-study, which included a sample of children with neurological disorders, showed that more than 90% of the children who received an ASD diagnosis according to the Diagnostic Interview for Social and Communication Disorders (DISCO) [29] were also scored above the 98^th^ percentile on the ASSQ by parents and/or teachers, corresponding to a sensitivity of 0.91 and specificity of 0.86 [28]. Furthermore, a factor analysis of the ASSQ items in the first wave of the BCS revealed a stable three-factor structure within both parent and teacher ASSQs. These factors were labeled “social difficulties”, “motor/tics/OCD”, and “autistic style” [30]. For validation purposes, the factors were correlated with the five subscales of the Strengths and Difficulties Questionnaire (SDQ): emotional problems, conduct problems, hyperactivity-inattention, peer problems, and prosocial behavior [31,32]. The ASSQ total score and factors showed the highest correlation with the SDQ peer problems subscale [30]. Another sub-study from the BCS on mental health problems in children with chronic illness found SDQ peer problems to be one of two areas scored particularly highly for children with neurological disorders [33]. These BCS findings motivated us to assess the utility of the ASSQ in detecting symptoms associated with ASD in children with neurological disorders.

The ASSQ is short, and unlike many other ASD screening instruments, it may be completed by parents and teachers alike. Multiple informants are considered to be important when screening children for psychiatric symptoms, as several studies have revealed only low to moderate agreement between raters [34-36]. For the ASSQ, the parent-teacher correlation was *r* = 0.66 in a clinical study of ASD assessment by Ehlers and collaborators [3], but only 15% of high-scorers were identified by both parents and teachers in the first wave of the BCS [37]. For ASD more generally, a Finnish population study found that only 24% of ASD cases were identified by both parents and teachers [38]. In a study of children with epilepsy, however, parent-teacher correlations for behavior ratings were moderate to high [39].

The aim of the present study was to assess symptoms associated with ASD in children with neurological disorders, as reported by parents and teachers on the ASSQ. Our study will thus be the first to investigate use of the ASSQ in this group of children. Based on results from earlier studies of children with neurological disorders, we expected to find the highest ASSQ scores and a higher frequency of children with a score associated with ASD in children with neurological disorders, as compared with control groups of children with other chronic illnesses or no chronic illness. Secondly, we wished to examine parent-teacher agreement on ASSQ-based assessments of children with neurological disorders, by looking at overall scores as well as three sub scores based on a factor analysis, and single item scores.

## Methods

### Participants

Data stem from the second wave of the BCS, an ongoing, longitudinal population based study of Norwegian children born in the years 1993–1995 (http://www.uib.no/bib). As the protocol and population of the BCS have been detailed elsewhere [40,41], the current paper will provide only a brief description. The first wave of the BCS was conducted in 2002. Parents and teachers of all children (9430 in total) attending second through fourth grade at elementary school (7–9 years of age) in any public or private school in the city of Bergen were asked to fill in a questionnaire, including the ASSQ. Informed consent to participate was provided by the parents of 7007 children. Based on the teacher ratings of 2544 children for whom parent ratings were not obtained, the impact of non-responses on estimates of mental health problems was examined in wave 1. Negligible non-response bias was found for mean scores and correlations, but logistic regressions indicated that children rated to have mental health problems by their teachers were less likely to participate. This mainly concerned children with moderate levels of symptoms [42]. The second wave was conducted in 2006. Parents and teachers of 5781 children, now in the fifth to seventh grades (11–13 years), participated alongside the children themselves by filling in a questionnaire, which again included the ASSQ. Compared to the wave one sample, the participants in wave two had somewhat lower mean ASSQ total scores as reported by parents and teachers, but differences were very small (*ES* =.05-.06). Ethnic diversity in this sample is expected to be minimal [43]. For more details about the second wave, see Hysing and collaborators [44]. The BCS was approved by the Regional Committee for Medical and Health Research Ethics Western Norway, and by the Data Inspectorate.

### Instruments

#### Chronic illness

As part of the second wave questionnaire, parents replied to a screening question of whether or not their child had a chronic illness or a disability. Parents who reported such an illness or disability as present, were asked to categorize it as a) asthma, b) epilepsy, c) diabetes, d) intellectual disability, or e) other illness. They were asked to specify if selecting the “other illness” category. Of the 5781 children, 496 were reported to have at least one chronic illness, including 99 children with a neurological disorder. Only “physical” conditions (and this included intellectual disability) were included, and an experienced pediatrician categorized the reported illnesses into subgroups. Children with more than one chronic illness were categorized according to a hierarchical order ranking “neurological disorders” above “other chronic illnesses”. The neurological disorder group included intellectual disabilities and related syndromes (40), epilepsy (30), migraine (20), cerebral palsy (10), hydrocephalus (6), neuromuscular disorders (3), brain tumor (2), neurofibromatosis (1), myelomeningocele (1), and moyamoya disease (1). The “other chronic illness” group included: asthma (234), allergy (134), eczema (36), diabetes mellitus (18), gastrointestinal disorders (17), skeletal disorders (15), sensory impairments (7), kidney disorders (4), endocrinological disorders (3), cardiovascular disorders (3), hemophilia (3), and rheumatism (3). Note that children may have more than one diagnosis, and that children with disorders co-existing with neurological disorders were classified as belonging to the neurological disorder group. It is also important to be aware that the presence of intellectual disability in children with neurological disorders is associated with increased risk of ASD [5,7,9,14,15,45]. We therefore controlled for the presence of intellectual disability (as reported by parents) in the present analyses.

### Autism spectrum symptoms

The ASSQ is designed to identify school-aged children who may need a more comprehensive evaluation due to suspected ASD. It was designed for completion by lay informants, and identical versions exist for parents and teachers. The ASSQ covers a wide range of symptoms predictive of a diagnosis within the autism spectrum, including difficulties with social interaction, verbal and non-verbal communication, restricted and repetitive behavior, motor clumsiness, and tics. It consists of 27 items scored on a three-point scale: not true (0), somewhat true (1), and certainly true (2). Possible scores range from 0–54, with higher scores indicating a greater symptom load [3]. Children exceeding the 98^th^ percentile on the parent and/or teacher reported ASSQ total score in the present sample were defined as high-scoring, indicating severe impairment according to a validation study by Posserud et al. [28].

The factor analysis from the first BCS wave (28), including parent and teacher ASSQ reports, was rerun on the full sample from the second BCS wave. The factor structure was confirmed with the following differences: two items, “Different voice/speech” and “Idiosyncratic attachment”, were not included in any factor in the analyses from the first wave, but now loaded clearly onto the second factor (motor/tics/OCD) for both parents and teachers, and were thus incorporated into this factor in the present study. The item “involuntary sounds” was accidentally omitted from the parent questionnaire in the second wave. It was therefore excluded from the teacher questionnaire in the present study. For more details regarding this factor analysis, see Posserud et al. [30].

### Missing data

When calculating the ASSQ total score, mean individual scores were inserted for missing items when 4/26 (15%) or fewer items were missing. ASSQ forms with more than four items missing were discarded from analyses of the ASSQ total score. Forms from 5129 parents (88.7%) and 5540 teachers (95.8%) were completed with fewer than four ASSQ items missing. When calculating the ASSQ factor scores, mean individual scores were inserted for missing items when only one item was missing. ASSQ forms with more than one item missing from any factor were discarded from factor score analyses. No missing items were substituted in the single item analyses. Data about intellectual disability and sex was present for 5116 (88.5%) and 5195 (89.9%) children, respectively. Due to the discrepancy between response rates for parents and teachers, and missing data regarding intellectual disability and sex, the number of participants varies across the present analyses.

### Statistical analyses

All analyses were performed using PASW Statistics 17. Descriptive statistics, Chi-squares and t-tests were used to explore sample characteristics. One-way between-groups analyses of covariance (One-way ANCOVA) were conducted to explore ASSQ scores reported by parents and teachers in the sample, with intellectual disability and sex as covariates. Due to uneven group sizes and violation of the homogeneity of variance, the Hochberg’s GT2 post hoc test was used to evaluate the results of the one–way ANCOVAs, and the significance level was set to p<.01. Effect sizes (Cohen’s *d*) were calculated and interpreted according to the guidelines of Cohen [46], in which a *d* value of 0.20 is small, 0.50 is medium, and 0.80 is large. Cross-tabs were used to calculate the frequency of children for whom the parent and/or teacher reported an ASSQ total score above the 98^th^ percentile. First, we calculated the frequency of children for whom *either* the parent *or* the teacher reported a score above the 98^th^ percentile; secondly, we calculated the frequency of children for whom *both* informant groups reported such a score. The same procedure was used to calculate the frequency of children scoring above the 80^th^, 90^th^, and 95^th^ percentiles. Bivariate correlations (Pearson’s *r*) were conducted to explore the agreement between the parent and teacher reported ASSQ scores in children with neurological disorders. These were interpreted according to the guidelines of Cohen (1988), in which an *r* value of .10 to .29 is low, .30 to .49 is moderate, and .50 to 1.0 is high. For analyses at the item level, scores of 0 (not true) and 1 (somewhat true) were collapsed, and the dichotomy *absence of behavior* (0, 1 = 0) and *presence of behavior* (certainly true; 2 = 1) was used. McNemar Chi-square tests were used to explore the relationship between item scores reported by parent and teachers for children with neurological disorders, using Cohen’s kappa [47] to quantify the level of inter-rater agreement. According to the guidelines presented by Fleiss [48], kappa values below .4 indicate poor agreement, values between .40 to .75 indicate fair to good agreement, and values above .75 indicate excellent agreement.

## Results

### Sample characteristics

The sample included 99 children with neurological disorders, 397 children with other chronic illnesses, and 5285 children with no chronic illness. Mean age across groups was 11.8 years. There were significantly more boys than girls in the groups with neurological disorders (*x*^2^(1) = 10.33, *p* = .001) and other chronic illnesses (*x*^2^(1) = 10.18, *p* = .001) as compared to in the no chronic illness group. The sex difference between the two chronic illness groups was non-significant (Table 1).

**Table 1:** Characteristics of the sample (N = 5781)

	**Neurological disorders**	**Other chronic illnesses**	**No chronic illness**
n (%)	99 (1.7)	397 (6.9)	5285 (91.4)
Boys (%)	63 (63.6)***	219 (55.3)***	2201 (46.8)
Age, *M* (*SD*)	11.82 (0.86)	11.77 (0.88)	11.80 (0.87)

***p<.001.

### The ASSQ total score

There was a statistically significant difference between the groups in terms of ASSQ total scores as reported by both parents (*F*(2, 5060) = 83.69, *p* = .0005, *d* = 0.36) and teachers (*F*(2, 5016) = 56.71, *p* = .0005, *d* = 0.30). This difference remained significant after controlling for intellectual disability (*F*(1, 5060) = 7.59, *p* = .01, *d* = 0.06) and sex (*F*(1, 5060) = 36.39, *p* = .0005, *d* = 0.17) for the parent reported ASSQ total score, and after controlling for intellectual disability (*F*(1, 5016) = 23.35, *p* = .0005, *d* = 0.14) and sex (*F*(1, 5016) = 170.96, *p* = .0005, *d* = 0.37) for the teacher reported ASSQ total score. Post-hoc tests showed that both parents and teachers reported children with neurological disorders to have significantly higher total scores than did children with other or no chronic illness (*p* < .01), with large effect sizes (*d* = .85-1.01). The mean difference in ASSQ total scores between children with other chronic illnesses and no chronic illness was non-significant for both informant groups (Table 2).

**Table 2:** Mean ASSQ-scores according to group and informant

	**Neurological disorders**	**Other chronic illnesses**	***d ***^**a**^	**No chronic illness**	***d ***^**b**^
**Parents, *M *(*SD*)**					
Total score	10.37 (9.70)**	3.33 (4.29)	.94	2.87 (4.14)	1.01
Social difficulties	5.36 (5.61)**	1.34 (2.35)	.93	1.08 (2.21)	1.00
Motor/tics/OCD	2.06 (3.11)**	0.35 (0.96)	.74	0.27 (0.96)	.78
Autistic style	2.95 (2.39)**	1.66 (1.91)	.60	1.53 (1.82)	.67
**Teachers, *M *(*SD*)**					
Total score	7.42 (8.40)**	1.97 (3.35)	.85	1.78 (3.55)	.87
Social difficulties	4.35 (5.29)**	1.11 (2.32)	.79	1.02 (2.34)	.81
Motor/tics/OCD	1.68 (2.81)**	0.25 (0.82)	.69	0.20 (0.84)	.71
Autistic style	1.38 (1.81)**	0.62 (1.14)	.50	0.55 (1.12)	.55

^a^ = Effect sizes (Cohen’s *d*) for the magnitude of the mean differences between children with neurological disorders and children with other chronic illnesses; ^b^ = Effect sizes (Cohen’s *d*) for the magnitude of the mean differences between children with neurological disorders and children with no chronic illness. **Significantly higher score in the neurological disorder group than in the two other groups at the *p*<.01 level.

### The ASSQ high scorers

The whole sample was used to identify children with a parent and/or teacher reported ASSQ total score above the 98^th^ percentile, resulting in a cut-off score of 17 for the parents and 14 for the teachers. Overall, 202 children (3.5% of the total sample) were identified as high-scorers by either a parent or teacher, while 31 (0.5% of the total sample) were identified as such by both informants. In the neurological disorder group, 31 children (31.3% of this group) were identified as high-scorers according to either the parent or the teacher, and 14 (14.1% of this group) were identified by both informants. The frequency of high-scoring children in the neurological disorder group was significantly higher compared to in the two other groups, as identified both by parents or teachers alone (*x*^2^(2) = 197.97, *p* = .0005), or by both informants (*x*^2^(2) = 338.79, *p* = .0005) (Table 3). As Table 3 shows, a higher frequency of children with neurological disorders also obtained an ASSQ total score equal to or above the 80^th^, 90^th^, and 95^th^ percentile, as compared to children in the two other groups.

**Table 3:** **Frequency of children within each of the three groups scoring above the 80**^**th**^**, 90**^**th**^**, 95**^**th**^**, and 98**^**th**^**percentile on the ASSQ total score according to a) *either *parent *or *teacher report; b) *both *parent *and *teacher report**

**a)**	**80**^**th**^**percentile**^**a**^	**90**^**th**^**percentile**^**b**^	**95**^**th**^**percentile**^**c**^	**98**^**th**^**percentile**^**d**^
**Neurological disorders**, n (%)	72 (72.7)***	57 (57.6)***	42 (42.4)***	31 (31.3)***
**Other chronic illnesses**, n (%)	150 (38.3)	81 (20.7)	47 (12.0)*	9 (2.3)
**No chronic illness**, n (%)	1572 (33.5)	880 (19.0)	400 (8.7)	162 (3.5)
**b)**	**80**^**th**^**percentile**^**a**^	**90**^**th**^**percentile**^**b**^	**95**^**th**^**percentile**^**c**^	**98**^**th**^**percentile**^**d**^
**Neurological disorders**, n (%)	51 (51.5)***	34 (34.3)***	23 (23.2)***	14 (14.1)***
**Other chronic illnesses**, n (%)	47 (11.9)**	22 (5.5)*	10 (2,5)	1 (0.3)
**No chronic illness**, n (%)	373 (7.5)	165 (3.3)	68 (1.3)	16 (0.3)

^a^ 80^th^ percentile = parent score of 5 and teacher score of 3; ^b^ 90^th^ percentile = parent score of 8 and teacher score of 5; ^c^ 95^th^ percentile = parent score of 12 and teacher score of 9; ^d^ 98^th^ percentile = parent score of 17 and teacher score of 14. ***Significantly higher than the two other groups at the p<.001 level; **significantly higher than the no chronic illness group at the p<.01 level; *significantly higher than the no chronic illness group at the p<.05 level.

### The ASSQ factor scores

The groups differed significantly in terms of their ASSQ social difficulties factor score as reported by both parents (*F*(2, 5053) = 77.65, *p* = .0005, *d* = 0.35) and teachers (*F*(2, 5009) = 43.57, *p* = .0005, *d* = 0.26). This difference remained significant after controlling for intellectual disability (*F*(1, 5053) = 30.65, *p* = .0005, *d* = 0.16) and sex (*F*(1, 5053) = 57.09, *p* = .0005, *d* = 0.21) for the parent reported social difficulties factor score, and after controlling for intellectual disability (*F*(1, 5009) = 24.23, *p* = .0005, *d* = 0.14) and sex (*F*(1, 5009) = 180.18, *p* = .0005, *d* = 0.38) for the teacher reported social difficulties factor score.

Further, the groups differed to a statistically significant level in their ASSQ motor/tics/OCD factor scores as reported by parents (*F*(2, 5060) = 74.56, *p* = .0005, *d* = 0.35) and teachers (*F*(2, 5015) = 54.19, *p* = .0005, *d* = 0.29). This difference remained significant after controlling for intellectual disability (*F*(1, 5060) = 14.27, *p* = .0005, *d* = 0.11) and sex (*F*(1, 5060) = 22.89, *p* = .0005, *d* = 0.14) for the parent reported motor/tics/OCD factor score, and after controlling for intellectual disability (*F*(1, 5015) = 44.76, *p* = .0005, *d* = 0.19) and sex (*F*(1, 5015) = 70.09, *p* = .0005, *d* = 0.24) for the teacher reported motor/tics/OCD factor score.

Finally, there was a statistically significant difference between the groups in the ASSQ autistic style factor score as reported by parents (*F*(2, 5061) = 29.51, *p* = .0005, *d* = 0.22) and teachers (*F*(2, 5024) = 17.21, *p* = .0005, *d* = 0.17). This difference remained significant after controlling for intellectual disability (*F*(1, 5061) = 6.99, *p* = .01, *d* = 0.06) and sex (*F*(1, 5061) = 3.64, *p* = .06) for the parent reported score, and after controlling for intellectual disability (*F*(1, 5024) = 0.12, *p* = .73) and sex (*F*(1, 5024) = 45.64, *p* = .0005, *d* = 0.19) for the teacher score. Post-hoc tests showed that both parents and teachers reported children with neurological disorders to have significantly higher factor scores than did children with other or no chronic illnesses (*p* < .01), with medium to large effect sizes (*d* = .50-1.00). The mean differences between the groups with other chronic illnesses and no chronic illness were all non-significant (Table 2).

### Informant agreement in children with neurological disorders

Correlations between parent and teacher ratings were high for their ASSQ total scores (*r* = .67) and social difficulties factor scores (*r* = .70), and moderate for the motor/tics/OCD (*r* = .53) and autistic style (*r* = .41) factor scores for children with neurological disorders. In contrast, the corresponding correlations for the group of other chronic illnesses were *r* = .43, *r* = .50, *r* = .33, *r* = .18; while for the group with no chronic illness they were *r* = .41, *r* = .46, *r* = .29, *r* = .17. All correlations were significant at the *p*<.01 level (two-tailed). Single-item analyses showed that parents of children with neurological disorders reported items as “certainly true” more often than did teachers, but this difference was statistically significant only on two items, i.e., “Bullied by other children” (*p* = .03) and “Insists on no change” (*p* = .03). In general, few children were co-identified by parents and teachers as presenting with a particular symptom, and kappa values were generally poor for most items except for “Lacks best friend”, “Lacks common sense”, “Poor at games, own goals”, and “Robotlike language”, for which kappa values were fair to good (Table 4).

**Table 4:** Percentage of children with neurological disorders identified by each informant and by both informants as presenting with a particular ASSQ item, and level of agreement reported as kappa values (n = 99)

**Social difficulties, %**	**Parents**^**a**^	**Teachers**^**a**^	**Co-id.**^**a**^	**Kappa**^**a**^
Lives in own world	14.1	17.2	6.1	.275
No social fit in language	16.2	14.1	5.1	.215
Lacks empathy	9.1	7.1	2.0	.185
Naïve remarks	11.1	6.1	3.0	.298
Deviant style of gaze	7.1	6.1	2.0	.259
Fails to make friends	12.1	11.1	3.0	.164
Sociable on own terms only	10.1	8.1	2.0	.145
Lacks best friend	21.2	17.2	10.1	.415
Lacks common sense	12.1	10.1	6.1	.489
Poor at games, own rules	18.4	19.4	10.2	.434
Bullied by other children	7.1*	1.0	1.0	.237
**Motor/tics/OCD, %**				
Different voice/speech	5.1	7.1	2.0	.291
Clumsy	15.2	17.2	7.1	.330
Involuntary movements	5.1	3.0	0.0	-.040
Compulsory repetitive	4.0	1.0	0.0	-.016
Insists on no change	8.3*	2.1	2.1	.379
Idiosyncratic attachment	7.1	5.1	2.0	.291
Unusual facial expression	1.0	4.0	0.0	-.016
Unusual posture	3.0	5.1	0.0	-.039
**Autistic style, %**				
Old-fashioned or precocious	19.2	9.1	3.0	.104
Eccentric professor	2.0	0.0		
Accumulate facts	10.1	4.0	1.0	.090
Literal understanding	7.1	4.1	1.0	.137
Robotlike language	2.0	2.0	1.0	.490
Idiosyncratic words	6.1	2.0	0.0	-.031
Uneven abilities	15.3	7.1	3.1	.194

Co-id. = percentage of children identified by both parents and teachers. ^a^Scores of 0 and 1 were collapsed. *Significantly higher at the p<.05 level.

## Discussion

### Summary of findings

In this population-based study of 11–13 year olds, children with neurological disorders obtained significantly higher parent and teacher reported ASSQ scores than did children with other chronic illnesses or no chronic illness. 14.1% of the children in the neurological disorder group obtained a score above the cutoff for positive ASD screening, as derived from combined parent and teacher ASSQ reports. The frequency of children identified with a score greater or equal to the 80^th^, 90^th^, and 95^th^ percentile cutoff points was higher than for children belonging to the two other groups. Overall, the agreement between parents and teachers of children with neurological disorders was moderate to high for the ASSQ total and factor scores, and poor for most of the single items.

### The ASSQ total score and high-scorers

The elevated ASSQ total score in children with neurological disorders is in accordance with the high rate of ASD symptoms found in studies of children with hydrocephalus [7,9] and epilepsy [11]. Although our sample represented a heterogeneous group including a range of neurological disorders, the combined parent and teacher ASSQ of 14.1% scoring above the 98^th^ percentile is in agreement with studies indicating that certain diagnoses included in our sample, for instance cerebral palsy [15], entail a heightened risk of ASD. Furthermore, our results showed that a large number of children with neurological disorders were rated as presenting with several ASD symptoms. A high frequency of children with neurological disorders was found above the 80^th^ percentile cutoff point on the ASD problem dimension in the total sample. These findings indicate that children with neurological disorders probably affect the percentile cutoffs for the total population by being overrepresented in the highest quartile.

### The ASSQ factor scores

The ASSQ scores of the neurological disorder group were elevated across all three factors as compared to the two other groups, i.e. in terms of social difficulties, motor/tics/OCD issues, and autistic style. The fact that the children with neurological disorders obtained the highest mean score and the largest effect size for the social difficulties factor score according to both informants, suggests that social difficulties are observed to be the most frequent symptoms in this group, and that the elevated total scores are only to a lesser extent affected by symptoms which may be more directly linked to the neurological disorders, such as motor difficulties. This result is in accordance with the study by Ekstrom and collaborators [14], in which impairment in social interaction and communication was the main problem in children with myotonic dystrophy and ASD.

### Informant agreement in children with neurological disorders

The study yielded high to moderate parent-teacher correlations on the ASSQ scores in children with neurological disorders, as in the study by Huberty and collaborators [39] which investigated informant agreement on behavior ratings in children with epilepsy. The correlation for the ASSQ total score in children with neurological disorders is comparable to the parent-teacher correlation of 0.66 found in the clinical study by Ehlers and collaborators [3]. In contrast, the correlations for children with other chronic illnesses and no chronic illness were mainly low or moderate, which is a general finding in reports on child psychiatric symptoms [34-36]. Furthermore, parents and teachers co-identified 45.2% of the ASSQ high-scorers in the neurological disorder group, which is higher than the equivalent rates for the two other groups, and higher than the 24% of ASD cases which were co-identified by parents and teachers in the study by Mattila and collaborators [38]. Altogether, these findings may indicate that parent-teacher agreement is higher in children with neurological disorders. On the other hand, the level of agreement between parents and teachers regarding the single items was generally poor, as in a previous BCS-study on items assessing ODD symptoms [36].

The relatively high correlation between the parent and teacher reported ASSQ total score may indicate that parents and teachers of children with neurological disorders have a shared understanding of the overall severity of the problem, but that, as indicated by the low level of agreement on the single items, they report different problems. This may be related to the different contexts in which they tend to observe the child. Parents more frequently see the child in a one-to-one setting, in which tics and compulsory behavior may be more easily observable, whereas teachers are better positioned to see the child interact with peers, and may thus be better able to identify social difficulties. Low parent-teacher agreement may also reflect differences in the understanding and interpretation of symptoms. For instance, the item “Bullied by other children” was reported as “certainly true” significantly more often by parents than by teachers. This might suggest that parents, more than teachers, perceive lack of friends or peer interaction as a type of bullying. Low agreement may also be partly explained by de facto differences in individual child behavior across settings. This underscores the general importance of obtaining information from both family and school settings when screening for problems associated with ASD. However, the high parent-teacher correlation on ASSQ scores in children with neurological disorders suggests that information from one setting may still be sufficient when assessing these children in a pediatric clinic.

### Strengths and limitations

The main strength of the present study is its population-based design. The total sample size and the size of the neurological disorder group increased the power and generalizability of the results. Another strength is the use of a validated instrument which included both parent and teacher reports.

This study has several limitations. First, chronic illness was assessed by parent reports, without medical verification of the diagnosis or its severity. Secondly, parent reports of intellectual disability were not validated by a formal test of intellectual function. Third, sensitivity to a prior ASD diagnosis may have biased the way in which parents and teachers responded to the ASSQ. Fourth, the overall participation rate was 70% in wave one and well above 50% in wave two. This attrition rate may have affected the representativity of the wave two participants, although the differences in mean ASSQ total scores between the wave one and wave two samples were very small. The lack of diagnostic assessment of ASD was another major limitation of the study. Parent and teacher reports on a screening questionnaire cannot replace a clinical validation of diagnosis. However, although no clinical validation study was performed in this second wave of the BCS, the rate of “clinically diagnosable” ASD in the children with a parent and/or teacher ASSQ total score above or equal to the 98^th^ percentile is likely to be high [28]. Nonetheless, caution should be applied in interpreting these results as indicating ASD rates, as firm conclusions require clinical confirmation.

### Clinical implications

The present findings of a high frequency of ASD related symptoms in children with neurological disorders emphasize the importance of clinicians having access to, and making skilful use of, screening instruments enabling them to recognize and identify problems which may pose a great challenge to the affected children and their families. The elevated scores on the social difficulties factor highlight the need for interventions targeting such difficulties in this group as a whole, and not only in children who meet criteria for a clinical diagnosis of ASD. As ASD share symptoms with other developmental/psychiatric disorders, such as ADHD, differential diagnostic evaluations are advised. The present study highlights for the first time the utility of the ASSQ to identify problems in children with neurological disorders. Its results call for future clinical studies of the children defined as “screen positives” for ASD in the present study.

## Conclusions

Parents and teachers of children with neurological disorders reported a high frequency of problems associated with ASD when completing the ASSQ. Awareness of these problems by parents, teachers as well as professionals within the health system may be crucial for the selection of successful treatment strategies. We therefore suggest that the ASSQ, or a similar instrument, should be included as part of the routine assessment of children with neurological disorders.

## Abbreviations

ASD: Autism Spectrum Disorder; ASSQ: The Autism Spectrum Screening Questionnaire; BCS: The Bergen Child Study.

## Competing interests

The authors declare they have no competing interests.

## Authors' contributions

HKR has been responsible for the data analyses and the writing of the manuscript. AJL designed and coordinated the study, supervised the data analyses and the writing process. MH has been responsible for creating data files, supervised the data analyses and commented on the written drafts of the manuscript. MP was responsible for the factor analysis and commented on the written drafts of the manuscript. CG has commented on the written drafts of the manuscript. All authors have read and approved the final manuscript.
